# Supplementary material for: Association between Dietary and Supplemental Antioxidants Intake and Lung Cancer Risk: Evidence from a Cancer Screening Trial
Source: Antioxidants (Basel). 2023 Jan 31;12(2):338. doi: 10.3390/antiox12020338 (PMC9952418; doi:10.3390/antiox12020338)
Supplement: Supplementary file 1 [file antioxidants-12-00338-s001.zip › antioxidants-2159421-supplementary.pdf]

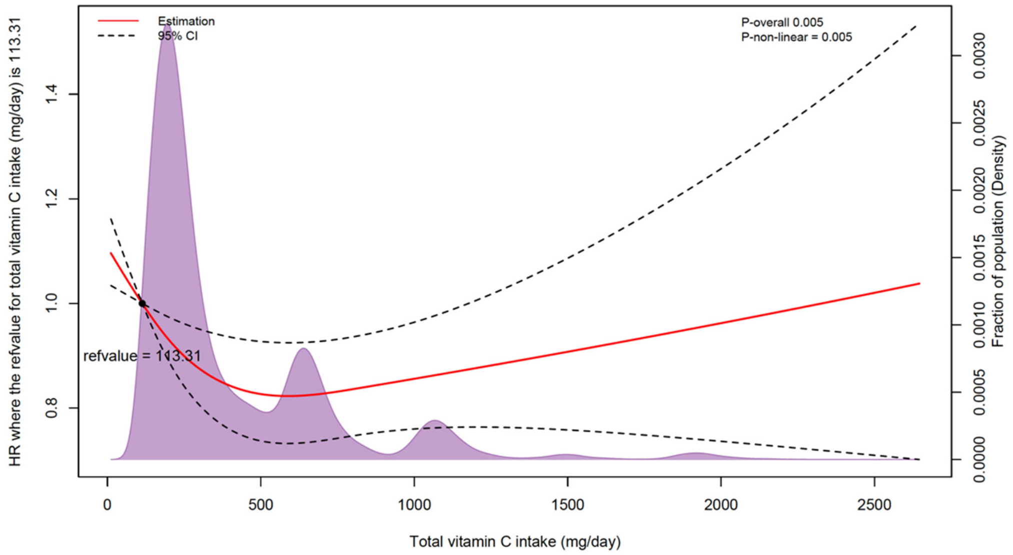

Figure S1. Dose-response curve for total vitamin C intake and lung cancer incidence

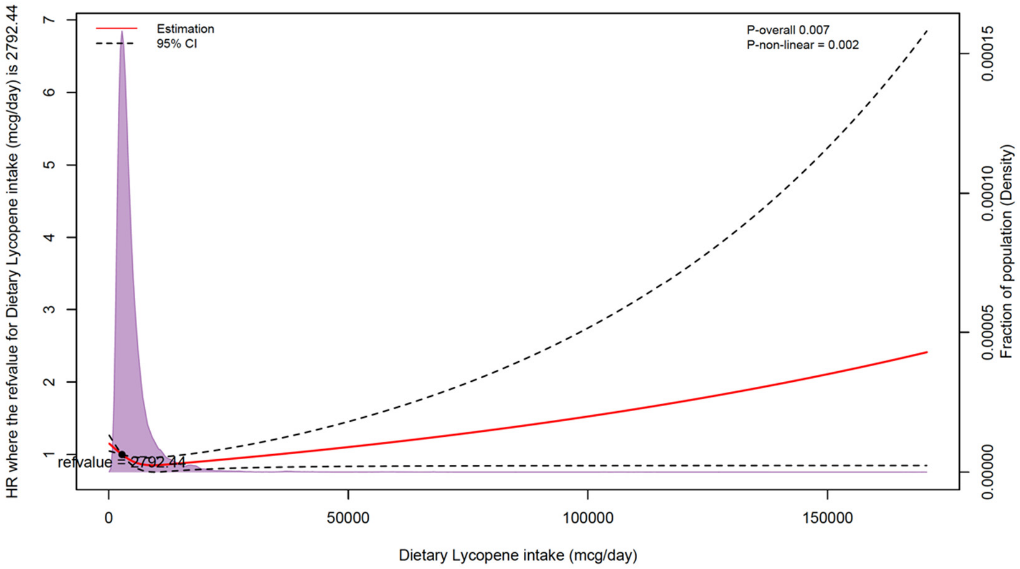

Figure S2. Dose-response curve for dietary lycopene intake and lung cancer incidence

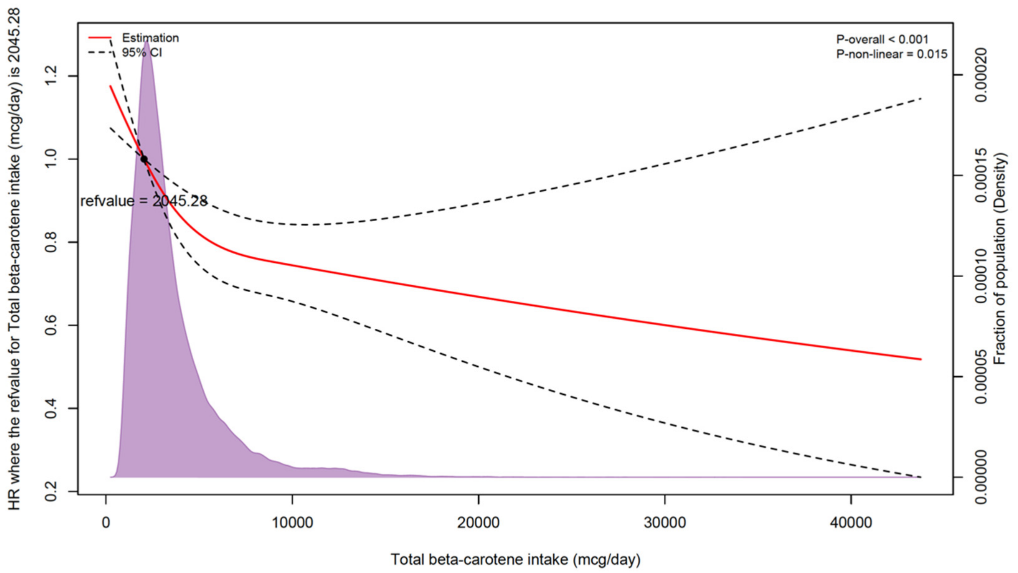

Figure S3. Dose-response curve for total β-carotene intake and lung cancer incidence

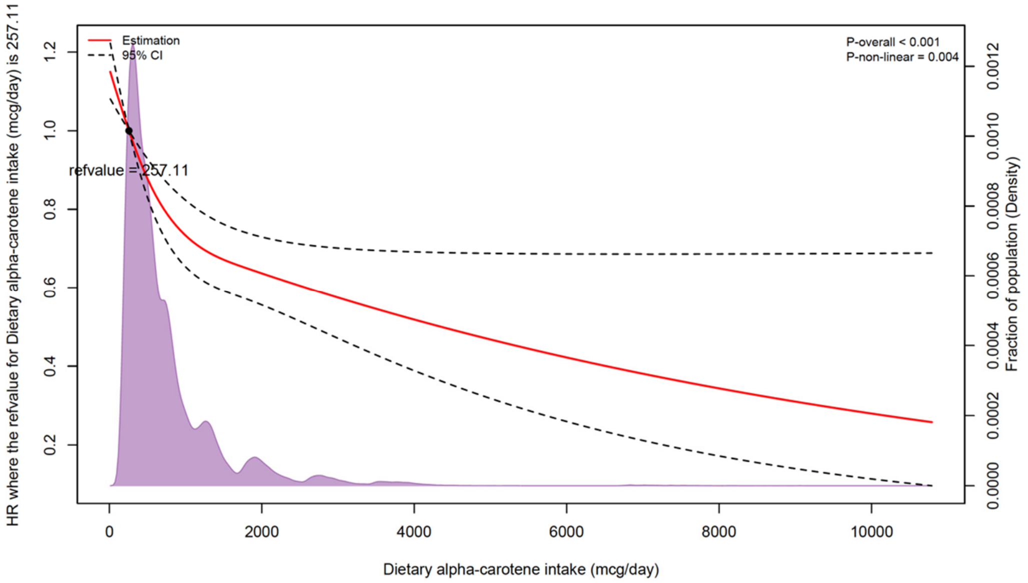

Figure S4. Dose-response curve for dietary α-carotene intake and lung cancer incidence

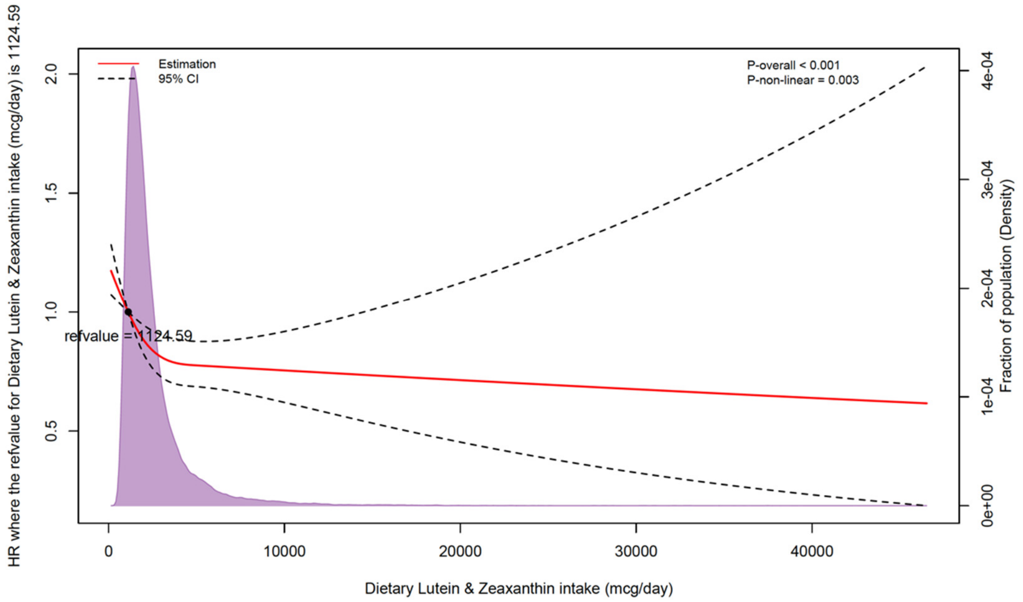

Figure S5. Dose-response curve for dietary lutein and zeaxanthin intake and lung cancer incidence

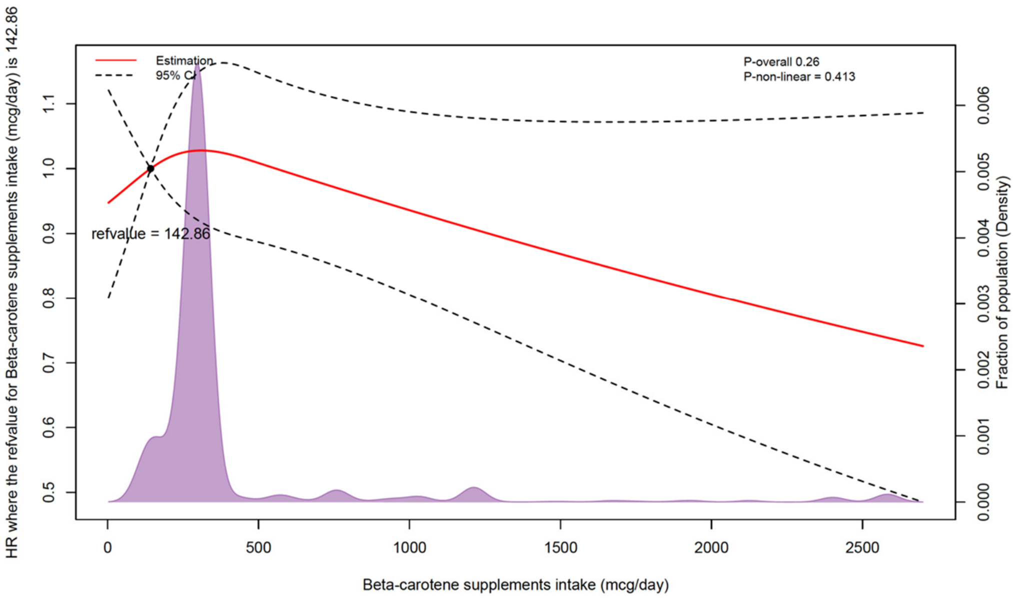

Figure S6. Dose-response curve for supplemental β-carotene intake and lung cancer incidence

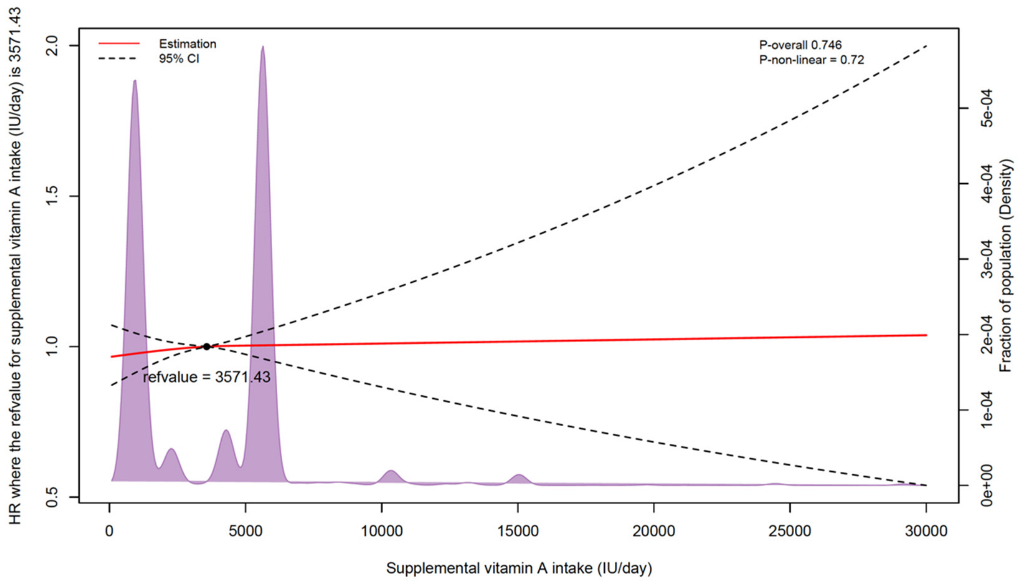

Figure S7. Dose-response curve for supplemental vitamin A intake and lung cancer incidence

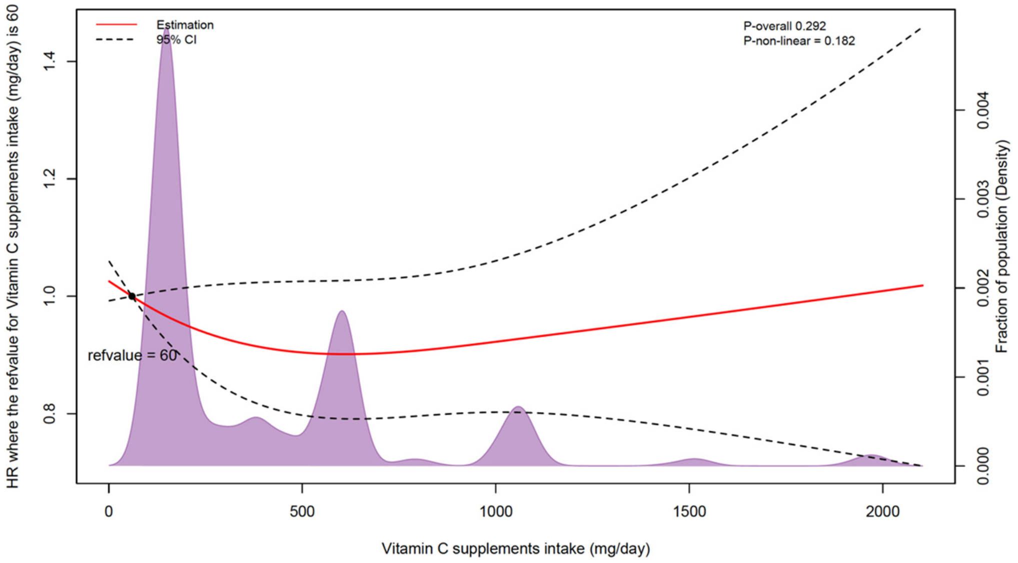

Figure S8. Dose-response curve for supplemental vitamin C intake and lung cancer incidence

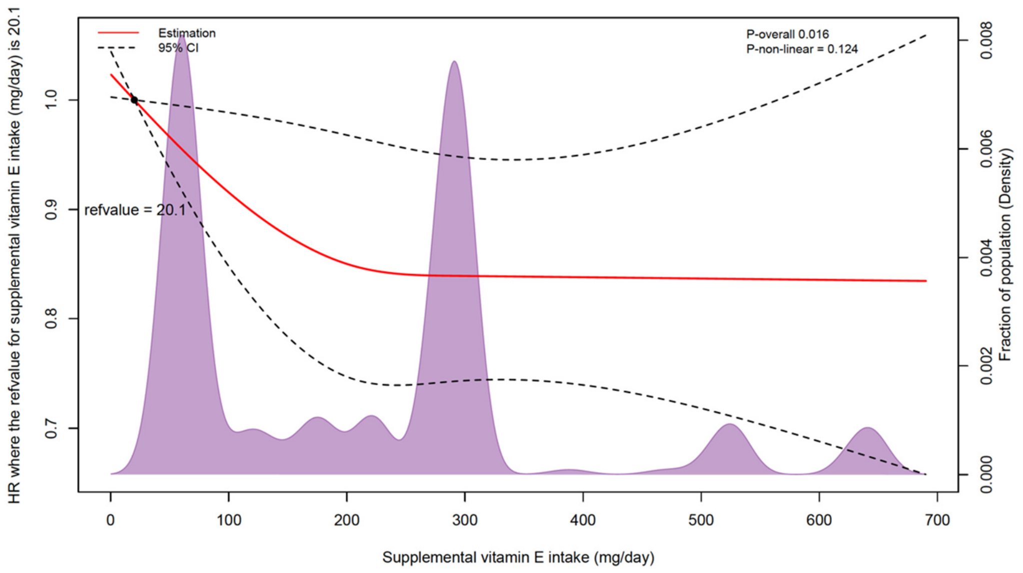

Figure S9. Dose-response curve for supplemental vitamin E intake and lung cancer incidence

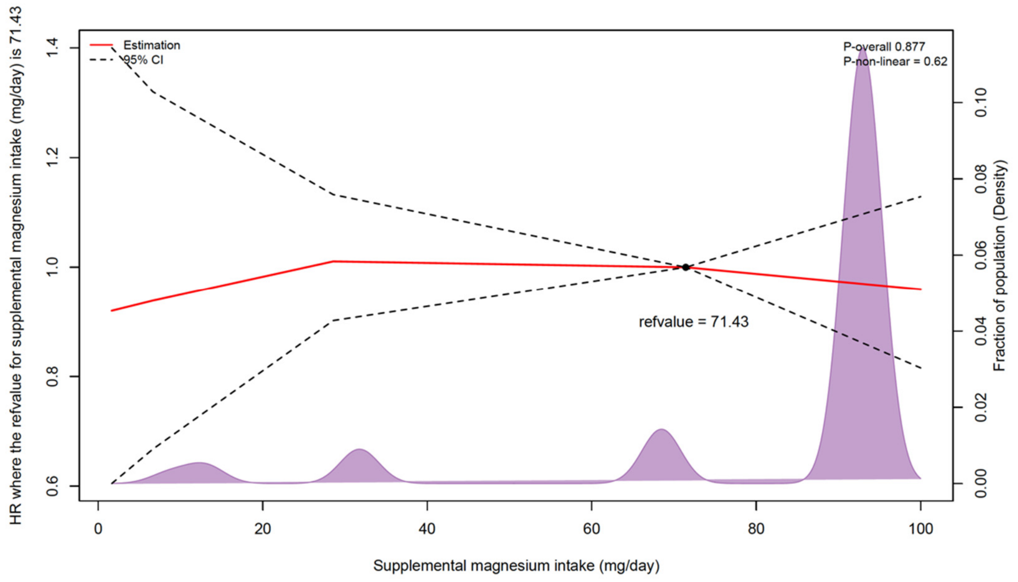

Figure S10. Dose-response curve for supplemental magnesium intake and lung cancer incidence

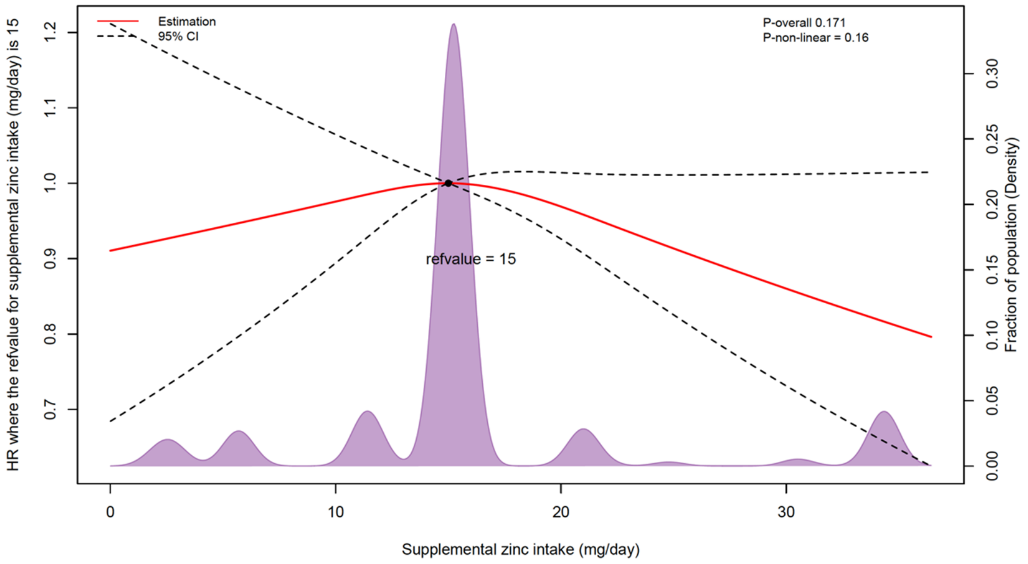

Figure S11. Dose-response curve for supplemental zinc intake and lung cancer incidence
